# Functional lupus anticoagulant testing in a large retrospective cohort of thrombosis patients with direct oral anticoagulants

**DOI:** 10.1038/s41598-020-69199-1

**Published:** 2020-07-22

**Authors:** Sara Reda, Anna Brügelmann, Jens Müller, Johannes Oldenburg, Bernd Pötzsch, Heiko Rühl

**Affiliations:** Institute of Experimental Haematology and Transfusion Medicine, University of Bonn, Venusberg-Campus 1, 53127 Bonn, Germany

**Keywords:** Diagnostic markers, Thromboembolism

## Abstract

Functional tests for lupus anticoagulants (LA) as part of a thrombophilia workup are commonly performed in patients under anticoagulant therapy that may interfere with assay results. There is no consensus on how these tests should be assessed in patients on direct oral anticoagulants (DOACs). In this retrospective cohort study, we analysed data from patients with a history of thrombosis in whom dilute Russell viper venom time (dRVVT), LA-sensitive aPTT, and solid phase assays for antiphospholipid antibodies (aPL) were performed (n = 3,147, thereof 588 on rivaroxaban, 144 on apixaban, 1,179 on other anticoagulant drugs). The dRVVT ratio was correlated with rivaroxaban (r = 0.30, *P* < 10^–4^) but not with apixaban plasma levels. The LA-sensitive aPTT/aPTT ratio showed no correlation with DOAC levels. Correspondingly, the rate of patients with abnormal dRVVT test was significantly higher (*P* < 10^–4^) under rivaroxaban (88%) than in thrombosis patients without anticoagulant medication (6%), independent from their aPL plasma levels. No isolated positive results of functional LA testing in patients on anticoagulants could be confirmed in repeated testing after discontinuation of the medication (n = 40). These data indicate that rivaroxaban should be discontinued before functional LA testing is performed. However, viable interpretation of these tests appears to be less affected in patients on apixaban.

## Introduction

Antiphospholipid antibody syndrome (APS) is an autoimmune disorder associated with thrombotic conditions and is regarded to be the most important acquired risk factor for vascular thrombosis, affecting approximately 10% of patients with a history of venous thromboembolism (VTE)^1,2^. Thus, screening for APS is included in the workup for a hypercoagulable state^3^. According to the international consensus criteria the diagnosis of APS is based on the combined presence of defined clinical manifestations as well as the persistent detection of antibodies directed against phospholipids, so called antiphospholipid antibodies (aPL)^4,5^.

Following the “Sidney criteria”, clinical manifestations include (1) one or more objectively confirmed clinical episodes of arterial, venous, or small vessel thrombosis and (2) complications of pregnancy, the latter including unexplained fetal death at or beyond the 10th week of gestation, premature birth before the 34th week of gestation because of eclampsia/pre-eclampsia or placental insufficiency, or three or more unexplained consecutive spontaneous abortions before the 10th week of gestation^4^. The persistent presence of either lupus anticoagulants (LA) or high immunoglobulin G (IgG) or immunoglobulin M (IgM) levels of anti-β2 glycoprotein I (aβ2GPI) or anti-cardiolipin (aCL) antibodies define the laboratory criteria for the diagnosis of APS^4^. LA are a heterogenous cluster of antibodies that prolong phospholipid-dependent clotting time in vitro^6^. The consensus recommendations include three different types of assays that complement each other in the detection of aPL: clot-based functional LA testing and solid phase assays to detect aCL and aβ2GPI. The patient is classified as having definite APS when at least one of the two clinical criteria is met and at least one of the two laboratory criteria is positive on two or more occasions at least 12 weeks apart^4^. The thrombotic and obstetric complications correlate most clearly with the presence of LA features^7–10^, making the detection of LA a most crucial step of screening for APS. There is, however, no complete agreement on how to best detect LA.

Primary criteria for LA detection include a prolonged coagulation time in a phospholipid-dependent clotting assay, the failure of a mixing test to correct this prolongation, the shortening of coagulation time in the presence of high phospholipid concentrations, and the absence of inhibitors for specific coagulation factors^11^. The International Society of Thrombosis and Haemostasis (ISTH)^12^ and the British Committee for Standards in Haematology (BCSH)^13^ advise the application of two tests based on different principles in LA detection. The first is the dilute Russell Viper Venom time (dRVVT) and the second is a LA-sensitive activated partial thromboplastin time (aPTT)^12,13^. This combination of tests evidently constitutes a sensitive and specific diagnostic strategy. However, with respect to the LA tests, there is no standardised reference method addressing the issue of quality control, and no available technique can detect all LA^6^.

Over and above these issues the tests currently used for the classification of definite APS have further limitations. All therapeutic anticoagulants have the potential to compromise functional LA testing whereas solid phase immunoassays for aPL remain unaffected. The impact of heparin and vitamin K antagonists (VKA) on functional LA testing has been well-recognised and implemented into the ISTH guidelines. To prevent misinterpretation, the guidelines recommend avoiding LA testing, if low molecular weight heparin (LMWH) has not been discontinued for at least 12 h or, in case of VKA, 1–2 weeks or until an international normalized ratio (INR) of 1.5 or less has been achieved^12^. Direct oral anticoagulants (DOAC), namely inhibitors of thrombin or activated coagulation factor X (FXa), have found increasing use in the treatment and prevention of VTE. Some concern has, however, been raised about false positive results of LA testing in patients taking these drugs. A possible interference of DOAC with LA testing was first reported in 2011 for patients on rivaroxaban enrolled in the EINSTEIN trial^14^. This initial report has been followed by several in vitro and ex vivo studies in small populations, stating that the dRVVT is the assay which is influenced the most, especially by rivaroxaban and dabigatran^15–19^. Controversial data have been reported regarding an effect of DOACs on aPTT-based assays^20,21^. Among the FXa inhibitors, apixaban has been reported to influence functional LA assays to a lesser extent than rivaroxaban and edoxaban^22–24^.

Several strategies are supposable and have been suggested to overcome the interference of anticoagulants with LA diagnostics, including postponing the investigation of LA until after discontinuation of continuous anticoagulant treatment. However, it is far from uncommon for samples from patients on anticoagulant drugs to be submitted for diagnostic LA testing. More practicable modifications of the strategy of postponed LA testing include blood sampling at trough levels of DOACs or LMWH or discontinuing these drugs for one or two days. However, there is paucity of data justifying such strategies.

## Results

### Study population and anticoagulant medication

Included over a 10-year period, the final study population consisted of 3,147 patients with a history of VTE or other venous or arterial thrombosis, thereof 2,419 classified as aPL^neg^, 477 as aPL^low^, and 251 as aPL^high^ based on the results of solid phase testing for aCL and aβ2GPI. The control group consisted of 1,395 individuals without a history of thrombosis or pregnancy complications, who did not show any abnormalities in thrombophilia screening. Demographic and clinical characteristics of the study population are shown in Table 1. The patients in the cohorts aPL^neg^, aPL^low^, and aPL^high^ did not show statistically significant differences regarding age or sex distribution. In comparison to these three cohorts, the individuals in the control group where younger, with a mean age of 32.3 years in comparison to 49.6–50.7 years (*P* < 10^–10^), and more frequently female (*P* < 10^–4^), as thrombophilia testing in these cases often was performed in the context of familial screening or contraceptive counseling. The mean dRVVT ratio was higher in the aPL^high^ group than in the aPL^low^ group, higher in the aPL^low^ than in the aPL^neg^ group, and higher in the aPL^neg^ group than in the controls (*P* < 10^–10^ for each comparison). The mean ratio of LA-sensitive aPTT/aPTT did not differ significantly between the aPL^neg^ cohort and the controls, but was higher in the aPL^low^ group than in these two cohorts (*P* < 10^–10^ each), and higher in the aPL^high^ group than in the aPL^low^ group (*P* < 10^–10^).

**Table 1:** Study population.

	Controls	aPL^neg^	aPL^low^	aPL^high^
Patients, n	1,395	2,419	477	251
Age, years	32.3 ± 16.6	49.6 ± 16.5	50.7 ± 16.5	50.6 ± 16.1
Sex (male/female)	320/1,075	1,050/1,369	186/291	99/152
History of VTE, n	–	1857	368	166
Other venous thrombosis, n	–	331	69	10
Arterial thrombosis, n	–	457	81	84
dRVVT ratio	1.02 ± 0.13	1.12 ± 0.24	1.21 ± 0.33	1.55 ± 0.77
Ratio: LAS aPTT/aPTT	1.04 ± 0.14	1.03 ± 0.08	1.07 ± 0.14	1.24 ± 0.45

LAS aPTT, Lupus anticoagulant sensitive aPTT; VTE, venous thromboembolism; dRVVT, dilute Russell viper venom time.

As shown in Table 2, a total of 1,911 patients in the study population were on anticoagulant medication, defined as any anticoagulant drug use within 14 days before blood sampling, thereof 1,429 in the aPL^neg^ cohort (59% of all patients in this group), 304 (64%) in the aPL^low^ cohort, and 178 (71%) in the aPL^high^ cohort. While the proportion of patients on anticoagulant medication in the aPL^low^ cohort did not differ significantly from the respective proportions in the aPL^neg^ cohort and in the aPL^high^ cohort, the latter proportion was significantly higher in comparison to the aPL^neg^ group (*P* = 6 × 10^–4^). Overall, the most frequently used anticoagulant drugs in the study population were VKA (41% of all patients on anticoagulant medication), followed by rivaroxaban (31%), LMWH (17%), and apixaban (8%). The remaining 3% of patients on anticoagulant medication were treated with dabigatran, edoxaban, or fondaparinux. They were not included in the further statistical analysis due to the small sample size. As shown in Supplementary Table S1 all but 9 patients applied the last dose of rivaroxaban, apixaban, or LMWH before blood sampling on the same day or the day before. No patient classified as “None or discontinued” regarding anticoagulant drug use had an INR of 1.4 or greater.

**Table 2:** Anticoagulant therapy.

Patients on anticoagulant drugs, n	aPL^neg^	aPL^low^	aPL^high^	Total
None or discontinued^a^	990	173	73	1,236
Rivaroxaban	459	92	37	588
Apixaban	103	26	15	144
Edoxaban	14	4	1	19
Dabigatran	21	4	4	29
VKA	575	119	84	778
LMWH	225	57	34	316
Fondaparinux	32	2	3	37

LMWH, low molecular weight heparin; VKA, vitamin K antagonist.

^a^Anticoagulant medication had been discontinued for at least 14 days.

### Effect of anticoagulant drugs on functional LA testing

**Figure 1:**
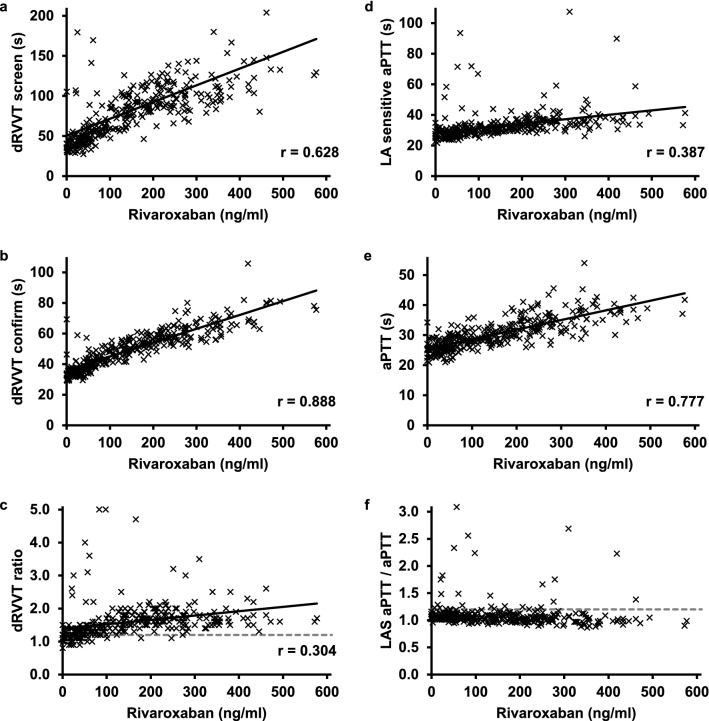
Correlation between rivaroxaban plasma level and functional LA test results. Measurement results of (**a**) dRVVT screen, (**b**) dRVVT confirm, (**c**) dRVVT ratio, (**d**) LA-sensitive aPTT, (**e**) aPTT, and (**f**) ratio of LA-sensitive aPTT/aPTT in n = 320 patients under anticoagulant treatment with rivaroxaban are shown. The intersected line indicates the upper reference limit of the functional LA tests of 1.2. The solid line indicates the Pearson correlation with the rivaroxaban plasma level, r indicates the correlation coefficient. LAS aPTT, Lupus anticoagulant sensitive aPTT; dRVVT, dilute Russell viper venom time.

**Figure 2:**
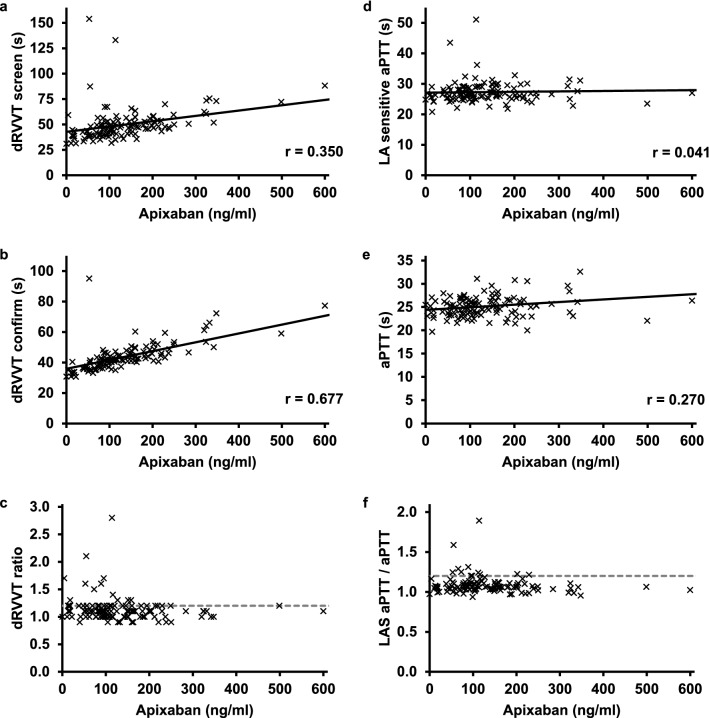
Correlation between apixaban plasma level and functional LA test results. Measurement results of (**a**) dRVVT screen, (**b**) dRVVT confirm, (**c**) dRVVT ratio, (**d**) LA-sensitive aPTT, (**e**) aPTT, and (**f**) ratio of LA-sensitive aPTT/aPTT in n = 119 patients under anticoagulant treatment with apixaban are shown. The intersected line indicates the upper reference limit of the functional LA tests of 1.2. The solid line indicates the Pearson correlation with the apixaban plasma level, r indicates the correlation coefficient. LAS aPTT, Lupus anticoagulant sensitive aPTT; dRVVT, dilute Russell viper venom time.

Data on plasma levels of rivaroxaban and apixaban were available for 320 and 119 patients, respectively. There was a mediocre to strong positive correlation between the plasma level of rivaroxaban and the dRVVT screen and confirm tests, and a clearly positive, albeit weak correlation of r = 0.304 (P < 10^–4^) between the dRVVT ratios and the rivaroxaban levels (Fig. 1a–c). The y-intercept of the correlation curve was 1.4, and there were 45 (14%) patients with a rivaroxaban level < 60 ng/ml who had a dRVVT ratio of > 1.20 (Fig. 1c), showing an interference of rivaroxaban with the dRVVT assay even at low plasma levels. The positive correlation between rivaroxaban level and dRVVT ratio remained statistically significant after exclusion of samples with rivaroxaban levels > 60 ng/ml from the analysis (r = 0.254, *P* = 0.011). The LA-sensitive aPTT and aPTT did also correlate positively with the plasma concentration of rivaroxaban but no significant correlation was observed for the ratio of LA-sensitive aPTT/aPTT (Fig. 1d–f). As shown in Fig. 2, there also was no significant correlation of dRVVT and LA-sensitive aPTT/aPTT ratios with plasma levels of apixaban. In patients on VKA, the dRVVT ratio, but not the ratio of LA-sensitive aPTT/aPTT increased with the INR, but the correlation (r = 0.111, *P* = 0.002) was very weak (Supplementary Fig. S1). In patients on LMWH, no significant correlation of the anti-factor Xa level with the dRVVT ratio and the ratio of LA-responsive aPTT/aPTT was observed (Supplementary Fig. S2).

The proportion of patients with abnormal results in the dRVVT test (a prolonged dRVVT screen and a dRVVT ratio of > 1.20) in the subgroups of the study population is shown in Table 3. In the control group 34 of 1,395 (2% of all individuals) showed abnormal results in the dRVVT test. In comparison to the controls, the proportion of abnormalities in patients with no or discontinued anticoagulant treatment was greater in the cohorts aPL^low^ and aPL^high^, with 14 of 173 (8%) patients (*P* = 6 × 10^–4^), and 23 of 73 (32%) patients (*P* < 10^–4^), respectively. There was no statistically significant difference between the controls and the aPL^neg^ cohort regarding the proportions of patients without anticoagulant treatment with respect to pathological results in the dRVVT test. As also shown in Table 3, the proportion of patients with abnormalities in the dRVVT test was significantly higher in patients on rivaroxaban or VKA in comparison to those without anticoagulant medication in the three patient groups aPL^neg^, aPL^low^, and aPL^high^, while there were no statistically significant differences between patients on apixaban, on LMWH, and patients without anticoagulant treatment. The proportion of patients on rivaroxaban with an abnormal dRVVT test remained significantly higher compared to those without anticoagulants when patients who took rivaroxaban on the day of the blood draw, and patients who took rivaroxaban one day before where analysed separately (Supplementary Table S2).

**Table 3:** Abnormal results of the dRVVT test depending on anticoagulant treatment.

Anticoagulant	Proportion of patients with abnormalities in the dRVVT, n			
	Controls	aPL^neg^	aPL^low^	aPL^high^
None or discontinued	2% (34)	4% (36)	8% (14)	32% (23)
Rivaroxaban	–	66% (301)*	79% (73)*	84% (31)*
Apixaban	–	7% (7)	12% (3)	33% (5)
VKA	–	19% (107)*	27% (32)*	64% (54)*
LMWH	–	6% (14)	17% (10)	38% (13)

Patients with a prolonged dRVVT screen and a dRVVT ratio > 1.2 were considered abnormal.

dRVVT, dilute Russell viper venom time; LMWH, low molecular weight heparin; NS, not significant; VKA, vitamin K antagonist.

*Significantly higher in comparison to the cohort with no or discontinued anticoagulants (*P* < 10^–4^ for all significantly different comparisons, calculated using the chi-squared test).

The proportion of patients with abnormal results in the LA-sensitive aPTT (a prolonged LA-sensitive aPTT and a ratio of LA-sensitive aPTT/aPTT > 1.20) in the subgroups of the study population is shown in Table 4. In the control group 47 of 1,395 (3%) individuals showed abnormal results. Compared with the controls, the proportion of abnormalities in patients with no or discontinued anticoagulant treatment was greater in the aPL^high^ group only, with 11 of 73 (15%) patients (*P* < 10^–4^). There were no statistically significant differences between the controls and the cohorts aPL^neg^ and aPL^low^ regarding the proportions of patients with pathological results in the LA-sensitive aPTT. As shown in Table 4, in comparison to the patients without anticoagulant medication the proportion of patients with abnormalities in the LA-sensitive aPTT was significantly higher in case of treatment with rivaroxaban only in the aPL^high^ group (*P* < 10^–4^). There was no significant difference between patients on the other anticoagulants and patients without anticoagulant treatment regarding the proportion of pathological results in the LA-sensitive aPTT.

**Table 4:** Abnormal results of LA-sensitive aPTT depending on anticoagulant treatment.

Anticoagulant	Proportion of patients with abnormalities in the LA-sensitive aPTT, n			
	Controls	aPL^neg^	aPL^low^	aPL^high^
None or discontinued	3% (47)	2% (19)	5% (9)	15% (11)
Rivaroxaban	–	2% (9)	5% (5)	54% (20)*
Apixaban	–	7% (7)	0% (0)	20% (3)
VKA	–	3% (15)	11% (13)	26% (22)
LMWH	–	5% (12)	16% (9)	32% (11)

Patients with a prolonged LA-sensitive aPTT and a ratio of LA-sensitive aPTT/aPTT > 1.2 were considered abnormal.

LMWH, low molecular weight heparin; VKA, vitamin K antagonist.

*Significantly higher in comparison to the cohort with no or discontinued anticoagulants (*P* < 10^–4^), calculated using the chi-squared test.

### Repeated testing of patients with abnormalities in functional LA assays

In the cohorts aPL^neg^ and aPL^low^, a total of 158 patients under anticoagulant therapy with abnormalities in functional LA testing were tested for a second time (Table 5). Although 104 of them still were on anticoagulant medication at the time of the repeated testing, 50% of these patients showed no abnormalities in the second analysis. Only three of the 54 patients (6%), in whom the anticoagulant medication was discontinued, tested positive again, and all these patients were from the cohort aPL^low^. In the cohort aPL^neg^, no abnormalities in functional LA testing could be confirmed in a second test after discontinuation of the anticoagulant medication.

**Table 5:** Repeated testing of LA positive patients using anticoagulant drugs in cohorts aPL^neg^, aPL^low^.

	Patients with anticoagulants at		LA positive^a^ with anticoagulants	
	1st testing	2nd testing	Continued	Discontinued
Rivaroxaban, n	83^a^	50	26/50 (52%)	2/33 (6%)
VKA, n	60^a^	44	18/44 (41%)	1/16 (6%)
Other, n	15^a^	10	8/10 (80%)	0/5 (0%)
Total, n	158	104	52/104 (50%)	3/54 (6%)^b^

dRVVT, dilute Russell viper venom time; VKA, vitamin K antagonist.

^a^Patients with a prolonged dRVVT screen and a dRVVT ratio > 1.2, and/or a prolonged aPTT and a ratio of LA-sensitive aPTT/aPTT > 1.2.

^b^In the cohort aPL^neg^ 0/40 patients with discontinued anticoagulant treatment were tested repeatedly positive for LA activity.

In the aPL^high^ cohort, data on repeated functional LA testing were available for 106 patients on anticoagulant therapy with abnormalities in initial testing. Functional LA testing for a second time was performed in most of these patients (n = 91) while continuing an anticoagulant therapy, as a large proportion of them had already been diagnosed with definite APS before. 79 (87%) of these patients with continued anticoagulant therapy also showed abnormalities in the second functional LA testing. Of the remaining 15 patients, in whom functional LA testing was performed after discontinuation of anticoagulant therapy for a second time, a similar proportion of 12 patients (80%) showed abnormalities.

## Discussion

In the present study we analysed the effect of anticoagulant medication on functional LA testing in the, to our best knowledge, largest real-world cohort of thrombosis patients to date. Among the different anticoagulant drugs used in this study population were all drugs or drug classes currently indicated for treatment or prophylaxis of VTE. However, only rivaroxaban, apixaban, LMWH and VKA were used in sufficiently large subpopulations to allow a reliable statistical analysis. This analysis focused on the questions, if and to what extent different anticoagulant drugs interfere with functional LA testing, which drugs should be discontinued before functional LA testing and how long before, and in which conditions results of functional LA testing can be viably interpreted.

The obtained data showed a clear correlation between plasma levels of rivaroxaban and the dRVVT ratio. This observation is in line with previous studies in smaller populations or in vitro analyses, in which an interference of rivaroxaban on functional LA testing has been shown^14–17,19,21,25–27^. Considering the calculated correlation coefficients rivaroxaban had the greatest impact on dRVVT results of all anticoagulant drugs evaluated in our study. The effect of rivaroxaban on the dRVVT was observed not only at high plasma levels of rivaroxaban but also at trough levels, which is consistent with previous reports^15,21,26^. A correlation of apixaban with the dRVVT ratio was not observed, which is in line with the results of two previous studies in smaller populations, one using the same reagents as were used in our study (LA1 and LA2 Screening reagent, Siemens)^23^, and the other one using different reagents (LA-screen and LA-confirm, Life Diagnostics)^27^. Conflicting with these reports and our observations, an interference of apixaban with the dRVVT was observed in two other studies using a different dRVVT test system (Diagnostica Stago)^17,26^. Differences between the reagents used in these studies and in the study presented here could possibly explain these discrepant findings. Differences in the affinities of rivaroxaban and apixaban to factor Xa have been suggested as an explanation of the varying effects of both factor Xa inhibitors on coagulation assays^28^. In patients on VKA we observed a significant, albeit rather weak, correlation between the INR and the dRVVT ratio, supporting current international guidelines that recommend discontinuing VKA before functional LA testing^12^. While a significant effect of LMWH on the dRVVT ratio or the ratio of LA-sensitive aPTT/aPTT was not observed, an influence of LMWH on functional LA testing cannot be excluded in individual patients. Therefore, these tests should be performed after discontinuation of LMWH for at least 12 h.

Although all anticoagulants assessed in the present study interfered with aPTT and LA-sensitive aPTT in a dose-dependent (or, in the case of VKA, INR-dependent) manner, no correlation with the ratio of LA-sensitive aPTT/aPTT was observed. In previous studies there was a great variation in the reagents used for aPTT-based functional LA testing as well as in interpretation algorithms. Consistent with our data, an effect of rivaroxaban on aPTT screening tests, but not on confirmatory tests or calculated aPTT-ratios, was observed in nearly all previous studies^14–16,19,21,27^. One previous study reported an interference of rivaroxaban and apixaban with aPTT-based LA-testing, but in this study the dRVVT was used to confirm initially abnormal results of the LA-sensitive aPTT^26^. There was one previous report of apixaban interfering with the aPTT-ratio (Diagnostica STAGO) in a small study population of 17 patients with atrial fibrillation^27^. The use of different reagents in this small study and in our study could possibly explain the different observations.

The study population was classified according to aPL levels quantified by solid phase assays, to account for the effect of “true” aPL on functional LA testing. In the absence of anticoagulant drugs, the proportion of patients with abnormal results in the dRVVT and the LA-sensitive aPTT increased with aPL plasma levels thus demonstrating an association between solid phase and functional assay results. Throughout the three subgroups with different plasma levels of aPL patients on rivaroxaban or VKA showed significantly higher frequencies of abnormal dRVVT results than patients with no or discontinued anticoagulant therapy. However, this was not true for patients on apixaban or LMWH. This observation further indicates that rivaroxaban and VKA interfere more with functional LA assays than apixaban and LMWH. Significantly higher frequencies of abnormal dRVVT results in comparison to patients without anticoagulant medication were also observed, when only patients, who took the last dose of rivaroxaban one day before blood sampling, were analysed. Thus, conducting functional LA testing at rivaroxaban trough levels, as has been previously suggested^19^, does not appear to be a reliable strategy to rule out interfering effects. In the subgroup of patients with high aPL levels there were also significantly more patients on rivaroxaban, who showed abnormal results in the ratio of LA-sensitive aPTT/aPTT, when compared to patients without anticoagulant treatment. This might indicate an effect of rivaroxaban on aPTT-based LA testing although no correlation between rivaroxaban levels and LA-sensitive aPTT/aPTT ratios was observed. Available data on repeated functional LA testing in the study population was analysed to assess, if repeated testing would be an adequate strategy to further clarify initially abnormal results. In 50% of the patients, in whom functional LA testing was repeated without discontinuing anticoagulant medication, and in nearly all patients, who discontinued the drugs, initially positive abnormal results could not be confirmed in the second analysis. These data strongly suggest that repeated functional LA testing with the intent to clarify initially abnormal results should only be performed after discontinuing potentially interfering anticoagulant drugs. Although the prothrombin time (PT) is less affected by LA than the aPTT due to the higher concentration of phospholipids in PT reagent, LA can occasionally manifest in a prolonged PT^29^ .As rivaroxaban and apixaban therapy may also be associated with a prolonged PT^24^, discontinuation of these drugs before laboratory testing would also improve detection of LA which cause a PT prolongation.

The major limitations of this study are the small size of the subgroup of patients, in which abnormal results of functional LA testing were validated after discontinuation of the anticoagulation medication, and the use of one specific test system for LA-sensitive aPTT and dRVVT, which precludes generalisation of the obtained results to other tests. Potential sources of bias or imprecision include the size of the overall study population, misclassification within the study population, and the performed laboratory analyses. To account for the potentially small sample size of some subpopulations an overall rather large cohort of thrombosis patients was included into this study. A selection bias might have been introduced by the postponed market availability of apixaban when compared to rivaroxaban, VKA, and LMWH, which were available throughout the whole study period. The performed analyses did not distinguish between different doses of drugs as this would have largely increased the number of sub-cohorts and reduced the statistical power of the study. The conducted laboratory analyses were covered by accreditation with the national accreditation body and were performed according to ISO standards.

In conclusion, the data obtained in this study confirm established knowledge of the influence of conventional anticoagulants on functional LA testing and broaden available data required to develop general recommendations for the diagnostic workup of APS in patients on DOACs. While rivaroxaban strongly affects the dRVVT test, our data provide evidence for a less pronounced interference of apixaban with functional LA testing. Thus, rivaroxaban should be discontinued for more than one day before functional LA testing. In contrast, dRVVT and LA-sensitive aPTT appear to provide a greater frequency of viable results when performed in patients on apixaban. However, as a false-positive result due to apixaban cannot be reliably excluded in the individual patient, functional LA testing without discontinuation of apixaban cannot be recommended.

## Methods

This retrospective cohort study was conducted at the Institute of Experimental Haematology and Transfusion Medicine, Bonn, Germany in patients who underwent LA and aPL testing in the thrombophilia outpatient clinic of our institution between 2008 and 2017. The study proposal was approved by the Institutional Review Board Ethics Committee of the Medical Faculty of the University of Bonn. Written informed consent was obtained from all participants in compliance with the declaration of Helsinki. The procedures followed were in accordance with institutional guidelines.

### Study population

Data on LA and aPL testing were retrieved from the database of the laboratory information system. In the above-mentioned 10-year period, a total number of 5,205 first-time referrals were identified, who received LA and aPL testing including LA-responsive aPTT, dRVVT, a solid phase screening assay for aPL covering aCL, and solid phase assays specific for aβ2GPI, and for anti-phospatidylserine-prothrombin antibodies (aPS/PT), respectively. Demographic and clinical data were extracted from the medical records of these patients by two independent reviewers. 3,147 of these 5,205 patients, who had a history of arterial thrombosis, VTE, or other venous thrombosis, were classified into three cohorts according to the results of the solid phase assays for aPL, (1) the cohort aPL^neg^ with levels of aCL and aPS/PT below their respective upper reference limits (URL) and aβ2GPI < 20 IU/ml, (2) the cohort aPL^high^ with levels of aCL IgG or IgM ≥ 40 IU/ml in the screening assay, or of aβ2GPI IgG or IgM ≥ 20 IU/ml (≥ 99th percentile of the reference interval), and (3) the cohort aPL^low^ with the patients not fulfilling the criteria for inclusion in cohort aPL^neg^ or aPL^high^. The control group was derived from the remaining 2,058 patients without a history of thrombosis, considering the following exclusion criteria: plasma levels of any aPL above the URL, a history of autoimmune diseases, therapy with anticoagulant or antiplatelet drugs, any abnormalities in a thrombophilia screening including deficiencies for antithrombin, protein C, and protein S, the factor V Leiden mutation, the prothrombin mutation 20210G>A, and, for women, pregnancy or puerperium, or a history of pregnancy loss. After exclusion of 663 patients due to the afore-mentioned exclusion criteria, the control group consisted of 1,395 patients.

### Laboratory analysis of blood samples

Blood samples were obtained from a venipuncture of a suitable arm vein using 21-gauge winged infusion sets (Sarstedt, Nümbrecht, Germany). After discarding the first 2 ml, blood was drawn into serum tubes and citrate tubes (10.5 mmol/l final concentration, Sarstedt). Serum and plasma samples were obtained by centrifugation (2,600×*g*, 10 min) within one hour after blood draw and stored at less than − 70 °C until assayed.

INR, aPTT, LA-sensitive aPTT, and dRVVT were performed using the BCS XP system (Siemens Healthcare Diagnostics, Eschborn, Germany) and corresponding reagents (Innovin, Actin FS, Actin FSL, LA1 and LA2 Reagent, Siemens Healthcare Diagnostic Products, Marburg, Germany). Actin FS and Actin FSL are both aPTT reagents with Actin FS having a higher phospholipid concentration than Actin FSL, which is used to determine the LA-sensitive aPTT. LA1 and LA2 both contain the venom of the Russell viper, which induces clotting by activation of coagulation factor X, and phospholipids at different concentrations. The screening reagent LA1 is used to determine a dRVVT “screen”, followed by determination of a dRVVT “confirm” using reagent LA2, which contains higher amounts of phospholipids. The results are expressed as ratio of LA-sensitive aPTT/aPTT, and of dRVVT screen/dRVVT confirm. Following the recommendations of the ISTH the principle of both assays is detection of prolonged phospholipid-dependent coagulation times in a first step, and correction of the prolonged coagulation times in the presence of high phospholipid concentrations in a confirmatory second step^11^. Intra-assay and inter-assay coefficients of variation (CV) of aPTT and dRVVT tests were calculated by measurement of 10 replicates of three blood samples in one run, and replicate measurement of 20 blood samples in three different runs on consecutive days. The intra-assay CV was 1.47% for the LA-sensitive aPTT/aPTT ratio, and 1.06% for the dRVVT ratio, while the inter-assay CV was 1.86% for the LA-sensitive aPTT/aPTT ratio, and 5.28% for the dRVVT ratio.

aCL, aPS/PT, and aβ2GPI were measured using enzyme immunoassays (Aeskulisa Phospholipid Screen GM and Serin-Prothrombin GM, Aesku.diagnostics, Wendelsheim, Germany; REAADS Anti-Beta2 Glycoprotein I (Aβ2GPI) IgM and REAADS Anti-Beta2 Glycoprotein I (Aβ2GPI) IgG, Diapharma Group, West Chester, OH, USA) which were performed on an automated ELISA processor (BEP 2000, Siemens Healthcare Diagnostics). The URL (97.5th percentile of the reference interval) of the enzyme immunoassays was calculated from normally distributed data obtained from a reference population of 120 healthy blood donors. The URL was 6.3 IU/ml for aCL IgG in the aPL screening test, 5.2 IU/ml for aCL IgM, 4.4 IU/ml for aPS/PT IgG, and 6.9 IU/ml for aPS/PT IgM. The lower reference limit (97.5th percentile) of the dRVVT ratio and the ratio of LA-sensitive aPTT/aPTT, both 1.2, were obtained in the same manner.

In samples from patients who received rivaroxaban, apixaban, or LMWH, the anti-FXa activity was determined using the Chromogenix Coamatic Heparin assay (Instrumentation Laboratory Company, Bedford, MA, USA). Calibration curves based on rivaroxaban or apixaban were utilized to calculate their plasma concentrations in samples from patients receiving these drugs.

### Data analysis

Data are generally presented as mean and standard deviation. All authors had access to primary data. Normality of data was tested using the Shapiro–Wilk test. An ANOVA test was performed, followed by an unpaired two-tailed Student t-test to compare data obtained in the separate cohorts of the study population. Generally, the chi-squared test was used for comparisons of frequencies, Fisher’s exact test was used for cell frequencies of 5 or below. *P* values ≤ 0.05 were considered significant. The Bonferroni method was applied to correct for multiple testing and was performed for 6 comparisons to compare each of the four cohorts with each other cohort, and for four comparisons to compare patients under the four different anticoagulant drugs rivaroxaban, apixaban, VKA, and LMWH with patients without anticoagulant medication. All calculations were performed using the XLSTAT statistical and data analysis solution software (Addinsoft, Boston, MA, USA).

## Supplementary information

Supplementary Figure S1.

Supplementary Figure S2.

Supplementary Figure Legends and Tables.

## Data Availability

The datasets generated during and/or analysed during the current study are available from the corresponding author on reasonable request.
