# Supplementary material for: Functional lupus anticoagulant testing in a large retrospective cohort of thrombosis patients with direct oral anticoagulants
Source: Sci Rep. 2020 Jul 22;10:12221. doi: 10.1038/s41598-020-69199-1 (PMC7376154; doi:10.1038/s41598-020-69199-1)
Supplement: Supplementary file 3 — Supplementary Figure Legends and Tables. [file 41598_2020_69199_MOESM3_ESM.docx]

**Functional Lupus Anticoagulant Testing in a Large Retrospective Cohort of Thrombosis Patients with Direct Oral Anticoagulants**

Sara Reda^1^, Anna Brügelmann^1^, Jens Müller^1^ Johannes Oldenburg^1^, Bernd Pötzsch^1^, Heiko Rühl^1^

^1^Institute of Experimental Haematology and Transfusion Medicine, University of Bonn, Bonn, Germany

**Legends to Supplementary Figures**

**Supplementary Figure S1** Correlation between INR and functional LA test results. Measurement results of **(a)** dRVVT screen, **(b)** dRVVT confirm, **(c)** dRVVT ratio, **(d)** LA sensitive aPTT, **(e)** aPTT, and **(f)** ratio of LA sensitive aPTT / aPTT in n=778 patients under anticoagulant treatment with vitamin K antagonists are shown. r indicates the Pearson correlation coefficient for the correlation with the INR. Abbreviations: LAS aPTT, Lupus anticoagulant sensitive aPTT; dRVVT, dilute Russell viper venom test.

**Supplementary Figure S2** Correlation between anti-Xa activity in plasma and functional LA test results. Measurement results of **(a)** dRVVT screen, **(b)** dRVVT confirm, **(c)** dRVVT ratio, **(d)** LA sensitive aPTT, **(e)** aPTT, and **(f)** ratio of LA sensitive aPTT / aPTT in n=316 patients under anticoagulant treatment with low molecular weight heparin are shown. r indicates the Pearson correlation coefficient for the correlation with apixaban plasma levels. Abbreviations: LAS aPTT, Lupus anticoagulant sensitive aPTT; dRVVT, dilute Russell viper venom test.

**Supplementary Tables**

**Supplementary Table S1** Interval between drug application and blood sampling

| **Time since last application** | **0 days*** | **1 day** | **2 – 14 days** |
| --- | --- | --- | --- |
| Patients on rivaroxaban, n | 363 | 218 | 7 |
| Patients on apixaban, n | 103 | 41 | 0 |
| Patients on LMWH, n | 201 | 113 | 2 |

*Drug application before blood sampling on the same day. Abbreviations: LMWH, low molecular weight heparin.

**Supplementary Table S2** Abnormal results of the dRVVT test depending on time of last rivaroxaban application

|  | **Proportion of patients with abnormalities in the dRVVT, n** | | | |
| --- | --- | --- | --- | --- |
| **Anticoagulant** | **Controls** | **aPL^neg^** | **aPL^low^** | **aPL^high^** |
| None or discontinued | 2% (34) | 4% (36) | 8% (14) | 32% (23) |
| Rivaroxaban at the day of blood draw | - | **81% (229)*** | **90% (54)*** | **95% (19)*** |
| Rivaroxaban at the day before blood draw | - | **41% (70)*** | **58% (18)*** | **63% (10)*** |

Patients with a prolonged dRVVT screen and a dRVVT ratio >1.2 were considered abnormal.

*Significantly higher in comparison to the cohort with no or discontinued anticoagulants (*P*<10^-4^ for all significantly different comparisons, calculated using the chi-squared test).
Abbreviations: dRVVT, dilute Russell viper venom time.
